# Obesity and the accelerated decline in total sleep time increases the self-reported diagnoses of diabetes

**DOI:** 10.3389/fendo.2025.1473892

**Published:** 2025-05-12

**Authors:** Lijing Yan, Huanhuan Sun, Yuling Chen, Xiaohui Yu, Jingru Zhang, Peijie Li

**Affiliations:** ^1^ Department of Endocrinology, The Second Affiliated Hospital of Xi ‘an Jiaotong University, Xi’an, China; ^2^ Department of Gastroenterology, The First Affiliated Hospital of Xi’an Jiaotong University, Xi’an, China; ^3^ Department of Respiratory Medicine, The First Affiliated Hospital of Xi ‘an Jiaotong University, Xi’an, China

**Keywords:** total sleep time, diabetes, time-dependent changes, longitudinal cohort study, joint model

## Abstract

**Introduction:**

The aim of this study was to investigate the relationship between obesity and the accelerated decline in Total Sleep Time (TST) and its potential impact on the self-reported diagnoses of diabetes.

**Methods:**

Our study addresses this gap by analyzing trends in a longitudinal cohort study conducted in China, using data from the China Health and Nutrition Survey (CHNS). Employing a joint model, inter-individual variability and intra-individual variability in TST, and its impact on self-reported diagnoses of diabetes were considered.

**Results:**

Our findings reveal that self-reported diagnoses of diabetes prevalence in China rose from 1.10% in 2004 to 3.06% in 2015, accompanied by a decrease in average TST from 8.12 to 7.80. With age, TST decreased by 0.01 per year. Among coffee or tea consumers, it decreased by 0.03, while alcohol users saw a decrease of 0.07. The obese group experienced a decrease of 0.05, the overweight group 0.03, and the normal weight group 0.01. Each 1-hour decrease in TST was associated with a substantial 3.61-fold increase in self-reported diagnoses of diabetes risk (95% CI: 2.92-4.44). Specifically, individuals with a higher baseline TST tend to experience smaller changes over time, whereas those with a lower baseline TST tend to experience larger changes.

**Discussion:**

For the obese, TST decreases at an accelerated rate which contributes to the risk of self-reported diagnoses of diabetes. The findings underscore the role of sleep loss in diabetes risk, with implications for public policy. Future research and interventions should emphasise the impact of sleep management, particularly on obesity and metabolic health, to develop more effective prevention and treatment strategies.

## 1 Introduction

A lack of sleep has become a common aspect of modern lifestyles (1). Extensive studies on the link between sleep deprivation and cardiovascular disease or mortality have classified sleep deprivation as an epidemic in some countries (2–4).However, there is still ongoing debate regarding the role of TST in causing diabetes (5). It has been established that the development of diabetes mellitus is influenced by a combination of genetic and environmental factors. Obese individuals and those with a family history of insulin sensitivity experience more erratic insulin sensitivity, and it is believed that sleep plays a role in this interaction. A Dutch study conducted on overweight and obese adolescents revealed that lower TST was associated with increased insulin sensitivity, particularly among those with a family history of non-insulin-dependent diabetes mellitus (6). Some experts argue that lack of sleep can affect blood sugar levels but may not necessarily lead to diabetes. Although in some studies, TST was recorded by some objective measures. Actigraphy has replaced Polysomnography (PSG) as the dominant tool in the study of sleep today. For studies with large populations, subjective sleep is still predominantly used due to the inaccessibility of equipment (Actigraphy or PSG). The existence of differences between subjective and objective sleep is then a matter of debate among scholars (7, 8). Several researches have now concluded that the objective TST results do not deviate from self-declared TST by more than half an hour, and the correlation coefficient between the two is 0.70 (9, 10). It appears that the credibility of self-reported remains acceptable, especially for large cohort studies in medically deprived areas (2, 11).

Given that early symptoms of diabetes are not readily apparent, blood testing is currently the only means available to identify and diagnose the condition. Consequently, early detection and screening for diabetes pose significant challenges. In epidemiological surveys, self-reported has emerged as a cost-effective approach with a high participation rate. Previous studies have confirmed the consistency between self-reports, drug utilization patterns, and medical records (12). Self-reported diagnoses of diabetes refers to patients being identified and informed of their condition by physicians using established diabetes diagnosis guidelines. While self-reported diagnoses of diabetes lacks objective measurement, it serves as a relatively reliable indicator of regional disparities in diabetes prevalence and undiagnosed cases, particularly in low- and middle-income areas where access to blood glucose testing and diagnosis is limited. It is worth noting that self-reported diagnoses of diabetes may underestimate the true prevalence due to the high occurrence of undiagnosed diabetes, but it remains a valid tool (13, 14). Furthermore, compared to other chronic diseases, self-reported diagnoses of diabetes exhibits the highest sensitivity and specificity (kappa: 0.84-0.76) (15). Its utilization in research is particularly advantageous in resource-constrained settings of low-income countries. Despite the limitations imposed by limited clinic access, self-reported diagnoses of diabetes generally aligns with the expected trends in the onset of diabetes. Moreover, when accurate data on the undiagnosed rate is unavailable, self-reported diagnoses of diabetes can serve as a corroborating indicator.

There is no consensus on the causal relationship between sleep duration and obesity in academic circles. Indian scholars have discovered that the risk of obesity or overweight in adults is associated with a short sleep duration (16). In pre-adolescent populations, scholars in Hong Kong reach similar conclusions (17). There is some modest evidence of a bilateral relationship. In a prospective study on middle-aged adults in the UK, prolonged sleep was associated with low BMI, while high BMI exacerbated shortened TST (18). Similarly, a US study found that sleep variability influenced adolescent obesity and cardiometabolic health (19). The majority of these studies focused on the average TST for the population and segmented it into various intervals, disregarding the differences between and within individuals. TST is influenced by factors such as gender and ethnicity, as it varies in levels between individuals (20–22). Within person level can change dramatically based on time, age, tobacco, alcohol, caffeine consumption, and weight status.

Sleep deprivation, obesity, and diabetes are significant public health concerns in today’s society (23). Obesity is widely recognized by the public as a risk factor for diabetes. It makes it impossible to ignore obesity and overweight as a factor in the study of the effect of TST on the survival of diabetes mellitus. So our study focuses on both the timing of self-reported diagnoses of diabetes and the possible longitudinal trajectory of change in the relationship between TST and weight status. To compensate for inefficiencies or biased results in separate analyses. We utilized a joint model of longitudinal and survival time outcomes to reveal the relationship.

## 2 Materials and methods

### 2.1 Study population

The CHNS is a stratified probability cohort study of the Chinese population that utilized a multistage, random cluster sampling strategy. It encompassed urban and rural areas in over 20 provinces and cities within China and included 15 ethnic groups, such as Man, Miao, Buyi, Tujia, and others. During the first stage of sampling, 2 cities and 4 counties were chosen in each province. In the second stage, a random selection of 2 urban and 2 suburban communities was conducted in each chosen city. In the third stage, a capital community and three rural villages were randomly selected in each chosen county, and 20 random families in each community were recruited. The CHNS commenced in 1989 and was tracked over nine waves in 1991, 1993, 1997, 2000, 2004, 2006, 2009, 2011, and 2015, with a survey population of almost 40,000 individuals overall. More information about the design, objectives, and survey methodology can be found on the CNHS homepage (https://www.cpc.unc.edu/projects/china). Since the earlier questionnaire did not have questions about sleep, our study was set to cover 2004 to 2015.

Our study enrolled participants who were between 18 and 65 years of age and completed questionnaires regarding their TST and diabetes diagnosis. Since the CHNS was conducted at different times, there were constantly new enrolled attendees and participants who were lost of follow-up. We conducted a current prevalence analysis with different time perspectives as cross-sectional data (as shown in **Table 1**). This dataset incorporates both survival data and longitudinal data traits. To compensate for the inefficiency of separate analyses or biased results, we conducted joint modeling of the two data types in order to explore their relevance. As shown in **Supplementary Figure S1**. We modeled the longitudinal measurements using linear mixed effects models and the survival data using the Cox proportional hazards model. Furthermore, we built a joint model to further analyze the dependencies and degree of association.

**Table 1:** Prevalence of self-reported diabetes in cross-sectional study.

		Cross-sectional data analysis for five waves				
		2004 (8190)	2006 (8010)	2009 (8255)	2011 (10770)	2015 (10014)
Diabetes (%)		90 (1.10)	104 (1.30)	171 (2.07)	330 (3.06)	337 (3.37)
Age ^Ʈ^		43.06 (12.03)vs.55.44 (7.05)**	44.09 (11.92) vs.53.76 (7.44) **	44.75 (12.15)vs.55.09 (7.27) **	45.62 (12.14)vs.55.69 (7.57)**	46.72 (12.02) vs.56.81 (6.80)**
Han vs. Etnic mhinority (%)		82 (1.15) vs.8 (0.77)	**98 (1.40)vs.6 (0.60)^#^**	153 (2.12) vs.18 (1.72)	313 (3.21)vs.17 (1.67)*	323 (3.61) vs.14 (1.32)**
Male vs. Female (%)		44 (1.11)vs.46 (1.09)	56 (1.45) vs.48 (1.15)	**97 (2.44) vs.74 (1.73)^#^**	164 (3.23) vs.166 (2.91)	**177 (3.77) vs.160 (3.01)^#^**
Urban vs. Rural residents (%)		49 (1.78) vs.41 (0.75)**	63 (2.38) vs.41 (0.76)**	96 (3.45) vs.75 (1.37) **	180 (4.10) vs.150 (2.35)**	172 (4.50) vs.165 (2.67)**
Education (%)	Low	44 (0.91)	**45 (1.06)^#^**	94 (2.02)	165 (3.07)	168 (3.42)*
	Middle	22 (1.21)	**25 (1.34)**	38 (2.13)	82 (3.35)	**96 (3.90)**
	High	7 (2.04)	**12 (2.51)**	13 (2.64)	40 (2.76)	**29 (1.88)**
	Unknown	17 (1.42)	**22 (1.56)**	26 (1.95)	43 (2.89)	**44 (4.00)**
Smoking vs. No (%)		29 (1.07) vs. 61 (1.11)	34 (1.34) vs.70 (1.28)	56 (2.15) vs.115 (2.04)	101 (3.08) vs.229 (3.06)	100 (3.72) vs.237 (3.24)
Alcohol vs. No (%)		**21 (0.74) vs. 69 (1.29) ^#^**	**26 (0.96) vs.78 (1.47) ^#^**	52 (1.77) vs.119 (2.24)	108 (2.77) vs.222 (3.23)	100 (3.43) vs.237 (3.34)
BMI (%)	NOB	34 (0.66)**	38 (0.77)**	58 (1.19) **	102 (1.74)**	100 (2.07)**
	Overweight	**35 (1.56)**	**43 (1.86)**	**75 (3.01)**	**142 (4.06)**	**144 (4.31)**
	Obesity	**17 (2.69)**	**22 (3.41)**	**37 (4.76)**	**86 (6.57)**	**90 (6.45)**
Coffee or tea vs. No (%)		**43 (1.39) vs.47 (0.92)^#^**	54 (1.92) vs.50 (0.96) **	71 (2.38) vs.100 (1.90)	161 (3.57) vs.169 (2.70) *	130 (4.25) vs.207 (2.98) **
PHD vs. No (%)		55 (11.43) vs.35 (0.45) **	56 (11.31) vs.48 (0.64) **	82 (15.33) vs.89 (1.15) **	155 (19.60) vs.175 (1.75) **	138 (20.72) vs.199 (2.13) **
TST (h)^Ʈ^		8.12 (1.16) vs.8.22 (1.56)	8.07 (1.14) vs.7.96 (1.25)	7.98 (1.13) vs.7.64 (1.22) **	7.83 (1.14) vs.7.60 (1.28)**	7.81 (1.09) vs.7.58 (1.21)**

^#^<=0.05.

^*^<=0.01.

**<=0.001.

^Ʈ^ Controls V.S. Cases.

Bold values indicate statistically significant results.

### 2.2 Outcomes and covariates

In this study, data on TST were collected from the CHNS Standard Questionnaire, which included the question, “How many hours each day do you usually sleep, including daytime and nighttime?” Participants were instructed to record their answers in hours.

To assess self-reported diagnoses of diabetes, the CHNS Standard Questionnaire was utilized, which included the question, “Has a doctor ever told you that you suffer from diabetes?” Those who responded affirmatively were classified as diabetic, while those who responded with unknown or no were classified as non-diabetic. We calculated Body Mass Index (BMI) by dividing the weight (kg) by the square of the height (m) based on physical measurements. We then graded BMI into three classifications according to the China obesity standard. BMI ≥ 28.0 was classified as obesity, BMI < 24.00 as normal or below (NOB), and overweight was situated between the two.

Parental history of diabetes (PHD) was determined by whether the father or mother had been diagnosed with diabetes at any time during the study period. Those with a reported diagnosis were classified as having a positive parental history, while those without a reported diagnosis were classified as having a negative parental history.

Covariates included in our study were age, sex (male, female), level of education (6–9 years for low, 10–12 for medium, and ≥ 13 for high, unknown), ethnicity (Han, other), residence area (urban, rural), tobacco use (yes or no), caffeine (coffee or tea) and alcohol consumption (yes or no).

### 2.3 Statistical analysis

First, we analyzed the prevalence and characteristics of self-reported diagnoses of diabetes using a cross-sectional study design across five different survey years. We used means (standard deviations) for TST and age that conformed to a normal or approximately normal distribution, and frequencies (percentages) for categorical variables. We all used the chi-square test to analyze the prevalence of self-reported diagnoses of diabetes among participants, considering factors such as gender, ethnicity, age, residence areas, education, smoking, drinking, coffee or tea, PHD, BMI, and TST. We employed one-way ANOVA to analyze trends for TST across survey years and to compare TST between diabetics and non-diabetics. Additionally, the Cochran-Armitage Test was used to analyze trends of diabetes prevalence over time.

Given the dynamic nature of the CHNS cohort, which allows for the continuous enrollment of participants, we have selected individuals who first joined the study in the years 2004, 2006, 2009, 2011, and 2015 as the subjects for our case-control analysis. The inclusion criterion was to utilize the baseline records of all participants upon their initial entry into the study for this analysis. Participants were excluded if their first record lacked sleep duration data, if they were outside the age range of 18 to 65, or if there was inconsistency in their self-reported diabetes status across follow-up assessments.

Subsequently, we established a cohort study by tracking participants who were initially undiagnosed with diabetes. We conducted descriptive statistics to summarize the basic characteristics of individuals included in the cohort study. (as shown in **Supplementary Table S1**)

In our longitudinal data analysis, we incorporated fixed effects to account for the influence of age and the interplay between BMI groups and observation time (Obstime) on TST. This approach allows us to explore how age and BMI categories dynamically interact with time to affect changes in TST. Furthermore, we acknowledged the potential impact of lifestyle choices on sleep by including covariates for habitual consumption of coffee or tea, alcohol intake, and smoking status. These factors are acknowledged for their capacity to significantly alter an individual’s sleep architecture and were thus controlled for within the model. To encapsulate the temporal dynamics and individual-specific fluctuations, Obstime was incorporated as a random effect. This strategy effectively integrates both systematic changes over time and the inherent randomness that shapes the individual’s sleep trajectory. The construction of a mixed linear model for TST represents our analysis’s objective of offering a comprehensive and nuanced portrayal of the multifaceted determinants of sleep patterns.


$$\begin{array}{c} \text{Level }-\;1:\;TST_{ij}\\ ={\text{β}}_{0j}+{\text{β}}_{1j}{\text{time}}_{ij}\times {\text{BMI}}_{ij}+\beta_{2j}{\text{AGE}}_{ij}+\beta_{3j}coffeeortea_{ij}\\ +{\text{β}}_{4j}{\text{ALCOHOL}}_{ij}+{\text{β}}_{5j}{\text{SMOKIND}}_{ij}+{\text{ε}}_{ij} \end{array}$$

$$\text{Level }-\;2:\;\beta_{0j}=\gamma_{00}+\mu_{0j},{\text{ β}}_{1j=}\gamma_{10}+\mu_{1j},\;{\text{ β}}_{2j=}\gamma_{20},{\text{ β}}_{3j=}\gamma_{30},{\text{ β}}_{4j=}\gamma_{40},{\text{ β}}_{5j=}\gamma_{50}$$

The term ${\text{β}}_{1j}{\text{time}}_{ij}\times {\text{BMI}}_{ij}$ the Level -1 equation is an interaction term. It allows us to understand how the effect of time on TST varies across different BMI groups. For example, in individuals with different BMI levels, the rate at which TST changes over time might be distinct. By including this interaction term, we can capture these complex relationships and provide a more accurate description of the data. Level -2, the random effects $\mu_{0j}$ and $\mu_{1j}$ are associated with the intercept $\beta_{0j}$ and the coefficient of the interaction term $\beta_{1j}$ respectively. The random intercept $\mu_{0j}$ accounts for the individual-specific differences in the baseline TST. Different individuals may have different starting points of TST even when all other covariates are the same. The random coefficient $\mu_{1j}$ , on the other hand, allows the relationship between time and TST to vary across individuals within each BMI group. This takes into account the inherent variability among individuals and helps us better fit the data and make more accurate predictions.

In order to address inquiries regarding the probability of self-reported diagnoses of diabetes and the time frame within which said reports occur as a consequence of variations in TST, we employed survival analysis, a powerful analytical tool. In our analysis, we considered a range of factors known to influence outcomes, including place of residence, ethnicity, gender, educational level, PHD, and BMI groups. These variables were integrated into a Cox proportional hazards model, which is an effective method for estimating the risk. Building upon this foundation, we advanced our analysis by constructing a joint model. This innovative approach enabled us to explore the intricate relationship between fluctuations in TST and the risk of self-reported diagnoses of diabetes across various BMI statuses. The joint model provides a more nuanced understanding of how sleep patterns interplay with other health indicators to affect the risk of self-reported diagnoses of diabetes.

We conducted statistical modeling and plotting using R4.2.1 with packages compareGroups,lme4, JM and ggplot2. We set p<=0.05 as the level of statistical significance in the study.

## 3 Results

**Figure 1:**
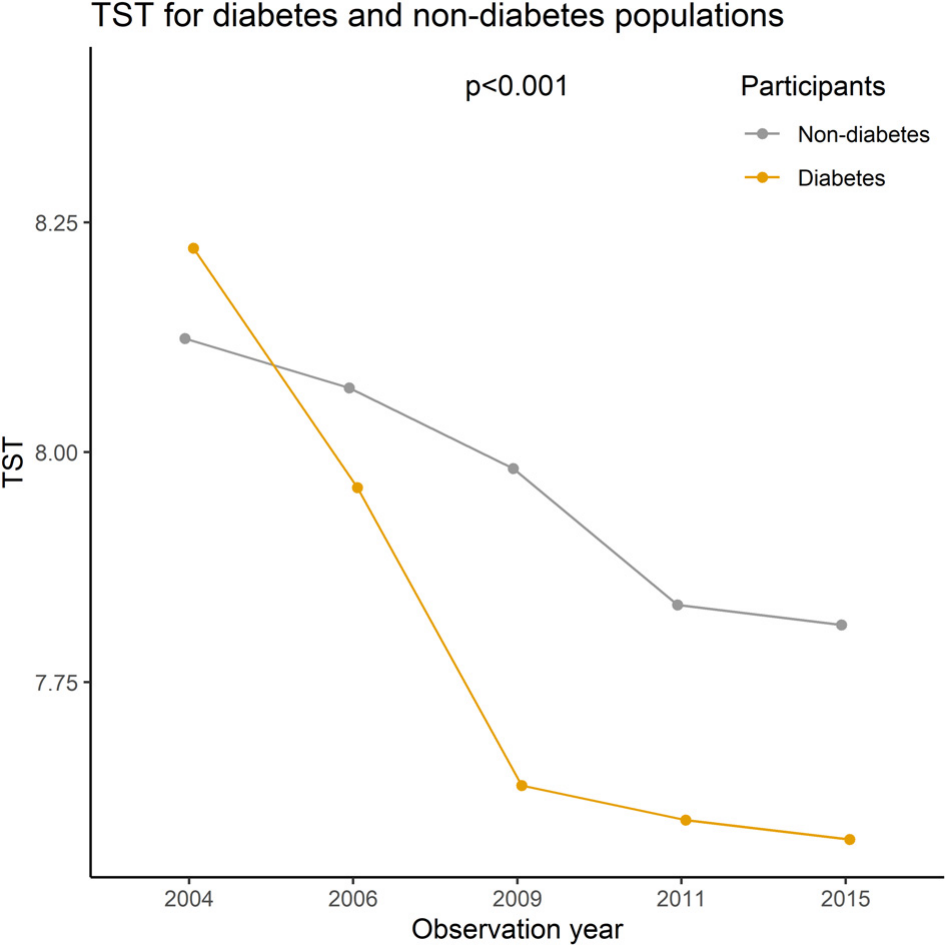
Mean TST for diabetes and non-diabetes in CHNS, 2004-2015.

The prevalence of self-reported diagnoses of diabetes showed a progressive increase from 1.10% in 2004 to 3.06% in 2015 (Z= -12.56, p<0.01). Meanwhile, the mean TST gradually decreased from 8.12 hours in 2004 to 7.80 hours in 2015 (F= 144.53, p<0.01). This decline was observed in both self-reported diabetes patients and non-diabetic participants, with TST being lower in self-reported diabetes patients than in non-diabetic participants (p<0.01) (refer to **Figure 1**). Prevalence differences were observed across various factors such as PHD, age, residence, BMI, and coffee or tea consumption group in almost all cross-sectional analyses. However, educational level, gender, and alcohol consumption showed variations in only some waves (as presented in **Table 1**). Baseline analysis results indicated that the risk factors for self-reported diagnoses of diabetes included PHD (OR= 14.2, p<0.001), overweight/obesity (OR= 2.64/3.98, p<0.001), age (OR= 1.12, p<0.001), Han ethnicity (OR= 1.98, p=0.002), urban residence (OR= 2.09, p<0.001), coffee or tea consumption (OR= 1.38, p=0.003), and a decrease of 1 hour in TST (OR= 1.26, p<0.001) (as shown in **Table 2**).

**Table 2:** A case-control study with initial cohort enrollment.

	Controls	Cases	*OR*	P.ratio	P.overall
	N=18463	N=376			
Sex					0.1
Female	9655 (52.29%)	180 (47.87%)	Ref.	Ref.	
Male	8808 (47.71%)	196 (52.13%)	1.19 [0.97;1.47]	0.09	
Age	41.53 (12.89)	55.43 (7.62)	1.12 [1.11;1.13]	<0.001	<0.001
PHD	0.06 (0.23)	0.46 (0.50)	14.2 [11.5;17.6]	<0.001	<0.001
BMI					<0.001
Normal	10826 (61.27%)	127 (34.79%)	Ref.	Ref.	
Overweight	5171 (29.26%)	160 (43.84%)	2.64 [2.09;3.34]	<0.001	
Obesity	1673 (9.47%)	78 (21.37%)	3.98 [2.97;5.29]	0	
Ethnicity					0.002
Han	16423 (88.95%)	354 (94.15%)	1.98 [1.32;3.15]	0.001	
Mhinority	2040 (11.05%)	22 (5.85%)	Ref.	Ref.	
Coffee or tea					0.003
No	12061 (65.33%)	217 (57.71%)	Ref.	Ref.	
Yes	6402 (34.67%)	159 (42.29%)	1.38 [1.12;1.70]	0.002	
Education					0.723
High	2140 (11.59%)	42 (11.17%)	Ref.	Ref.	
Low	9636 (52.19%)	187 (49.73%)	0.99 [0.71;1.40]	0.934	
Middle	4606 (24.95%)	101 (26.86%)	1.11 [0.78;1.62]	0.556	
Unknown	2081 (11.27%)	46 (12.23%)	1.13 [0.74;1.72]	0.583	
Alcohol					0.272
NO	12143 (65.77%)	258 (68.62%)	Ref.	Ref.	
YES	6320 (34.23%)	118 (31.38%)	*0.88 [0.70;1.09]*	0.249	
Smoking					0.271
NO	12838 (69.53%)	251 (66.76%)	Ref.	Ref.	
YES	5625 (30.47%)	125 (33.24%)	1.14 [0.91;1.41]	0.248	
Residence areas					<0.001
Rural	11120 (60.23%)	158 (42.02%)	Ref.	Ref.	
Urban	7343 (39.77%)	218 (57.98%)	2.09 [1.70;2.57]	<0.001	
TST ^ƛ^	8.01 (1.16)	7.71 (1.42)	1.26 [1.15;1.37]	<0.001	<0.001

^ƛ^ Decreasing by 1 unit.

**Figure 2:**
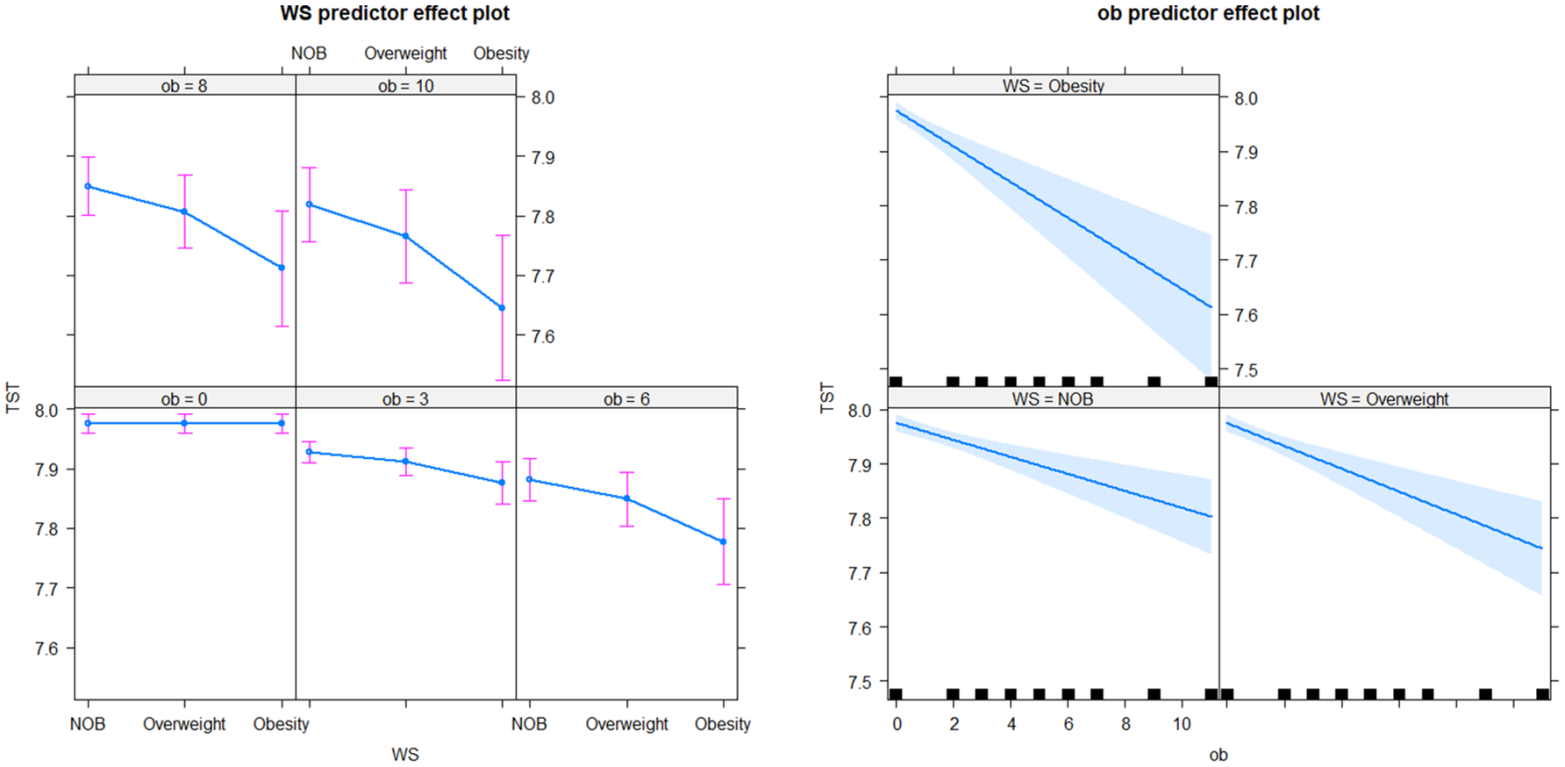
TST by the interaction effect between BMI and Observation time.

**Figure 3:**
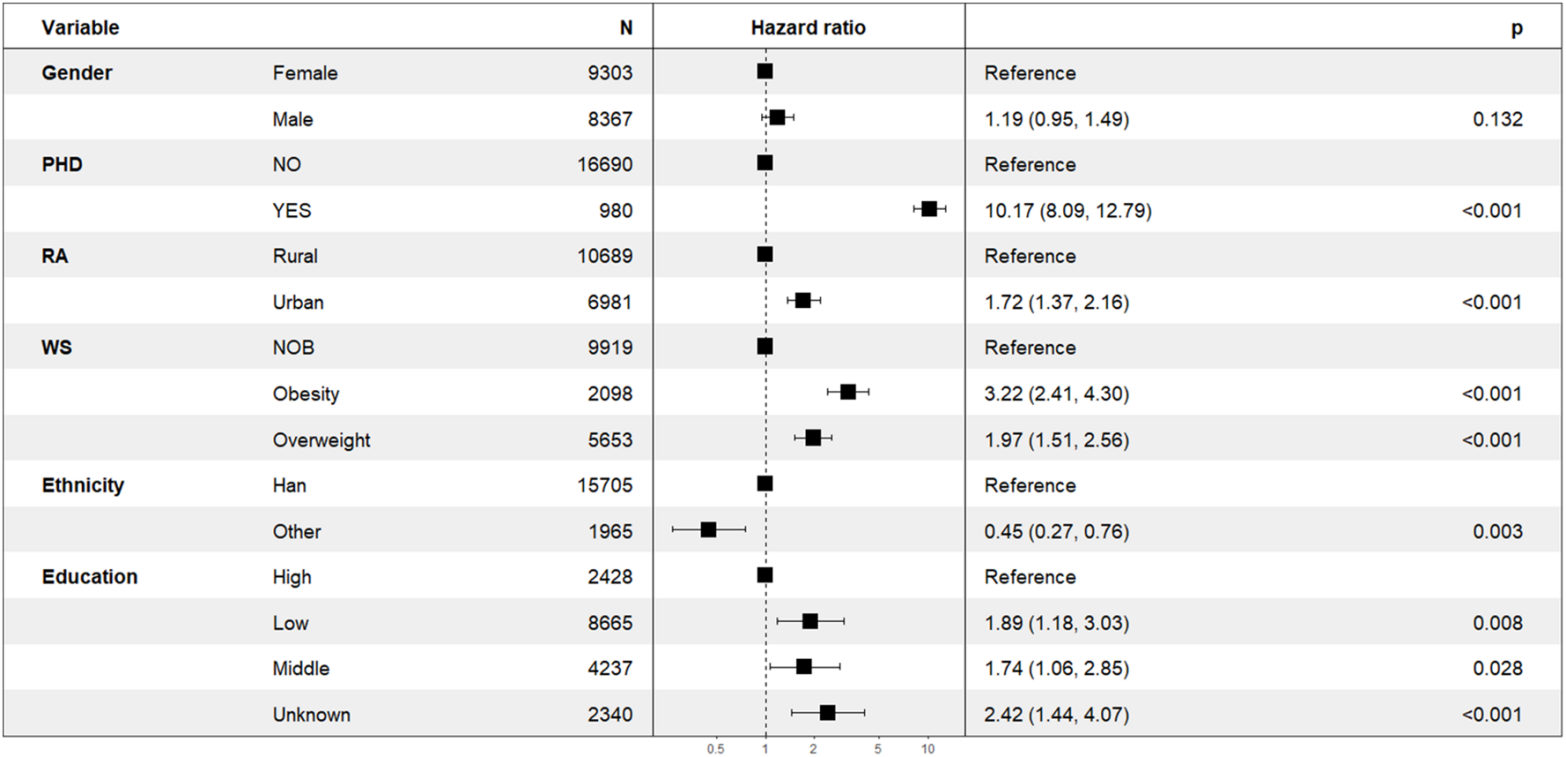
Cox regression forest plot of self-reported diabetes survival data.

In the longitudinal sub-model, the intercept, age, BMI× Obstime, coffee or tea consumption, and alcohol consumption were all found to be statistically significant. TST progressively decreased with age, coffee or tea consumption, and alcohol consumption. The interaction between different BMI groups and time was also statistically significant, suggesting that the trend of TST over time varied across BMI groups (as shown in **Figure 2**). The deceleration was fastest in obese individuals and second fastest in overweight. As for the random effects section, the correlation coefficient of -0.65 between intercept and slope indicated a negative relationship between baseline TST and the magnitude of change in the outcome variable. Specifically, individuals with a higher baseline TST tend to experience smaller changes over time, whereas those with a lower baseline TST tend to experience larger changes. In the survival sub-model, PHD, BMI groups, residence, ethnicity, and literacy were all found to be statistically significant (as shown in **Figure 3**).

In the longitudinal process, TST decreased on average by 0.01 with each annual increase in age. Furthermore, TST was lower by an average of 0.03 in the group consuming coffee or tea compared to the non-consuming group, and by 0.07 in the alcohol-consuming group compared to the non-alcohol group. Among the BMI groups, TST decreased on average by 0.05 in the obese group with each follow-up visit, by 0.03 in the overweight group, and by 0.01 in the NOB group. In the event process, the Hazard Ratio (HR) for self-reported diagnoses of diabetes was 1.84 (95% CI: 1.40-2.41) in the overweight group and 2.81 (95% CI: 2.07-3.80) in the obese group compared to the normal and underweight group. The HR was 1.99 (95% CI: 1.18-3.34) for secondary education and 2.15 (95% CI: 1.31-3.53) for lower education compared to tertiary education. Urban residents had a HR and 95% CI for self-reported diagnoses of diabetes of 0.65 (0.52-0.83) compared to rural residents, and 0.46 (0.27-0.78) for ethnic minorities compared to Han Chinese. The relative HR for the PHD group compared to the non-PHD group was 9.95 (95% CI: 7.88-12.57), and for females compared to males, it was 0.87 (0.69-1.10). The joint model with diabetic self-reports revealed an association coefficient of -1.28 (-1.49 – 1.07) and a 95% confidence interval. Specifically, each 1-hour decrease in TST was associated with a substantial 3.61 (2.92-4.44) fold increase in the risk of self-reported diagnoses of diabetes (see **Table 3**).

**Table 3:** Joint model for TST and risk of self-reported diabetes in CHNS.

		Linear mixed-effects sub-model			Longitudinal Process	Joint Model		
Fixed effects		Value	95%CI	P-value		Value	95%CI	P-value
Intercept		8.54	8.49-8.59	<0.001		8.56	8.51-8.60	<0.0001
**BMI× Observation time(Obstime)**	NOB**×** Obstime	-0.02	-0.02–0.01	<0.001		**-0.01**	**-0.02–0.01**	**<0.0001**
	Overweight**×** Obstime	-0.02	-0.03–0.01	<0.001		**-0.02**	**-0.03–0.02**	**<0.0001**
	Obesity**×** Obstime	-0.03	-0.05–0.02	<0.001		**-0.05**	**-0.06–0.04**	**<0.0001**
Age		-0.01	-0.01**–**0.01	<0.001		-0.01	-0.01**–**0.01	<0.001
Coffee or tea		-0.03	-0.05**–**0.00	0.03		-0.03	-0.05**–**0.00	0.02
Smoking		0.02	-0.01**-**0.04	0.30		0.01	-0.02**-**0.04	0.37
Alcohol		-0.07	-0.10**–**0.04	<0.001		-0.07	-0.10**–**0.04	<0.001
**Random effects**		**StdDev**	**Corr**			**StdDev**	**Corr**	
Intercept		0.51				0.51		
Obstime		0.05	-0.65			0.05	-0.65	
Residual		1.03				1.03		

		Survival sub-model						
		HR	95%CI	P-value	Event Process	HR	95%CI	P-value
Gender	Female	0.84	0.67-1.05	0.13		0.87	0.69-1.10	0.24
History of parental diabetes	Yes	10.17	8.09-12.79	<0.001		9.95	7.88-12.57	<0.0001
Education	Low	1.89	1.18-3.03	0.008		2.15	1.31-3.53	0.0025
	Middle	1.74	1.06-2.85	0.028		1.99	1.18-3.34	0.0093
	Unknown	2.42	1.44-4.07	0.001		2.64	1.53-4.55	0.0005
**BMI**	Overweight	1.97	1.51-2.56	<0.001		**1.84**	**1.40-2.41**	**<0.0001**
	Obesity	3.22	2.41-4.30	<0.001		**2.81**	**2.07-3.80**	**<0.0001**
Ethnicity	Other	0.45	0.27-0.76	0.003		0.46	0.27-0.78	0.004
Residence areas	Rural	0.58	0.46-0.73	<0.001		0.65	0.52-0.83	0.0004
**TST**						**3.61**	**2.92-4.44**	**<0.001**

Bold values indicate statistically significant results.

The symbol "×" represents the multiplicative interaction term.

## 4 Discussion

Our study provides compelling evidence that the average TST is decreasing while the self-reported rate of diabetes continues to increase in China between 2004 and 2015. TST was affected by age, coffee and tea consumption, drinking habits, and obesity level. PHD, lower education level, heavier obesity, urban dwellers, and Han Chinese had a higher risk of the disease than the control group. We specifically analyzed the time-dependent variability of TST among individuals with diverse BMI states. As the obese state with-in individual, TST decreases at an accelerated rate. Our results showed that a 1-hour decrease in TST was associated with a 3.61-fold increase in the risk of self-reported diagnoses of diabetes. These findings provide a new vision for diabetes intervention through sleep manipulation in different populations. They also highlight the impact of overweight and obesity status on the temporal relationship between sleep and self-reported diagnoses of diabetes.

It’s an indisputable fact that modern people are sleeping less and less. The reduction in TST as individuals age aligns with previous literature (24). Our study identified an average TST decrease of 6 minutes per decade. This differs slightly from the SIESTA data, which suggests a decline of 8–10 minutes per decade. The discrepancy in measurement methods, with our study using self-assessment on an hourly scale and SIESTA employing PSG measurements on a minute scale, could explain this slight disparity (25). Caffeine and alcohol consumption habits may lead to an average reduction in TST by 2 and 4 minutes, respectively. Caffeine intake prolongs the time taken to fall asleep and reduces the overall duration of sleep (26, 27). These findings have been consistently supported in regions where tea or coffee consumption is prevalent (28, 29). The amount of caffeine consumed and the timing of consumption play a crucial role in influencing TST (30, 31). Public awareness regarding the potential negative effects of alcohol on sleep is often lacking, and alcohol is frequently used as a sleep aid. However, alcohol actually leads to a decrease in TST during the latter half of the night and an increase in both the frequency and duration of awakenings (32). The study conducted in the Stonington, Massachusetts area, female participants who consumed alcohol experienced a 19-minute decrease in TST compared to those in the placebo group, whereas the difference was not significant among male participants (33). NHIS data from the United States (2004-2015) confirmed that the prevalence of short sleep was higher in drinkers than in the non-drinking group (34). Our research findings are inconsistent with those of Shabana Masood, as we did not find any evidence that smoking reduces sleep duration (35). It is possible that the discrepancy arises from the fact that their study focused solely on adult males and categorized smoking into three levels.

We identified a parallel relationship between obesity and TST. Obesity state led to a decline in TST at a rate of half an hour per decade. Similarly, overweight status resulted in a decrease in sleep of 12 minutes over a 10-year period. Our findings align with Koolhaas et al.’s longitudinal data analysis, revealing a link between high BMI and reduced sleep duration over time (18). The trend of TST varied across different BMI groups, with the obese group showing the most significant decrease, followed by the overweight group.

In an increasing number of regions and countries, the general public has been urged not to ignore and underestimate sleep problems (23, 36). Also, some experiments have been conducted on sleep interventions and it is believed that sleep manipulation is feasible. Several studies have shown that it is feasible to prolong sleep using simple non-pharmacological interventions, including behavioral interventions and health education (37, 38). The effectiveness of these interventions has also been demonstrated in randomized controlled studies. At the same time, some studies have confirmed that sleep manipulation can change an individual’s food intake and dietary preferences. These include reduced intake of free sugars, fats, and carbohydrates and reduced overall appetite and desire for sweet and savory foods (38, 39).Cognitive-behavioral therapy with sleep manipulation is more effective than cognitive-behavioral therapy alone in weight loss in the overweight and obese population by Logue et al. (40)

In recent years, the intricate relationship among obesity, reduced sleep duration, and diabetes has emerged as a prominent research focus in the field of metabolism. Scholars have been exploring integrative theoretical models, emphasizing the bidirectional causal or mediated causal mechanisms among these factors. Individuals with overweight often report a shortened sleep duration (41), and this is also corroborated by animal models, which demonstrate that obesity can lead to insomnia (42). Research has found that the “Raptin” reveals potential molecular mechanisms underlying the connection between sleep and obesity, providing valuable insights into this complex relationship (43). Additionally, models have been proposed suggesting that obesity acts as a mediator in the relationship between sleep and diabetes (44).

Recent evidence suggests that sleep duration and diabetes can be explained by mechanisms such as inflammatory responses, reduced insulin sensitivity, and increased appetite. However, the effect of the TST (time-dependent variable) and obesity on the temporal relationship of diabetes mellitus is unclear. While previous research has adjusted for BMI and obesity indicators when examining the correlation between TST and diabetes, it may not fully explain the dynamic fluctuation of TST in relation to weight status and its impact on diabetes (23). To address this gap, our study employs a joint model of longitudinal and survival data to investigate diabetes self-reports in the CHNS. This novel model incorporates longitudinal TST measurements, obesity grading, and other relevant factors. The tendency for the higher baseline TST is likely to be small, and conversely the tendency for the lower baseline TST to be large. The higher rank of obesity, the faster TST declined over time, with a 3.6-fold increase in self-reported diagnoses of diabetes risk for each hour of decrease in TST. Despite the lack of direct evidence linking extended sleep duration to the prevention and treatment of diabetes, there are indications that alleviating sleep deprivation may improve insulin sensitivity (23, 45). Moreover, as mentioned earlier, sleep manipulation may have a positive influence on obesity. Therefore, sleep manipulation could potentially serve as a significant intervention strategy to decrease diabetes prevalence and alleviate the burden of disease in our modern society.

In conclusion, our findings indicate suggest a significant increase in the prevalence of self-reported diagnoses of diabetes and a significant decrease in TST among Chinese adults over the past decade. Furthermore, our analysis revealed that the time-dependent variability of TST among individuals with diverse BMI states and found that a 1-hour decrease in TST was associated with a 3.61-fold increase in the risk of self-reported diagnoses of diabetes. For the obese, TST decreases at an accelerated rate, thereby increasing the risk of self-reported diagnoses of diabetes. These initiatives provide new ideas to further explore the dynamic causal relationship and specific mechanisms of the three (obesity, reduced sleep duration, diabetes). It could also serve as effective policy measures for diabetes control. Despite studies confirming the effectiveness of metformin in preventing diabetes in obese people under the age of 60, the American Diabetes Association now recommends metformin as a viable option for diabetes prevention (46). However, it is important to note that this recommendation complements, rather than replaces, the emphasis on lifestyle interventions as the cornerstone of diabetes prevention strategies (47). Sleeping interventions are likely to be included.

Our study has several limitations that need to be acknowledged. First, the self-reported nature of sleep duration or diabetes in the survey may have introduced recall bias in the interviews. However, previous studies have demonstrated the reliability and accuracy of self-reported measures compared with objective measures. Additionally, self-reporting remains a cost-effective method with high participation rates. Second, the study measured and analyzed TST in hours, potentially overlooking small yet significant differences. Third, the TST in our study represented the combined value of lunch break duration and night sleep duration, preventing separate consideration of their effects and hindering the accurate assessment of their relative and independent contributions to diabetes. The results need to be used with more caution, as there are also studies showing that napping for more than 30 minutes may increase the risk of T2DM (48).

## Data Availability

Publicly available datasets were analyzed in this study. This data can be found here: https://www.cpc.unc.edu/projects/china.
