# Supplementary figures and images for: Obesity and the accelerated decline in total sleep time increases the self-reported diagnoses of diabetes
Source: Front Endocrinol (Lausanne). 2025 May 12;16:1473892. doi: 10.3389/fendo.2025.1473892 (PMC12104077; doi:10.3389/fendo.2025.1473892)

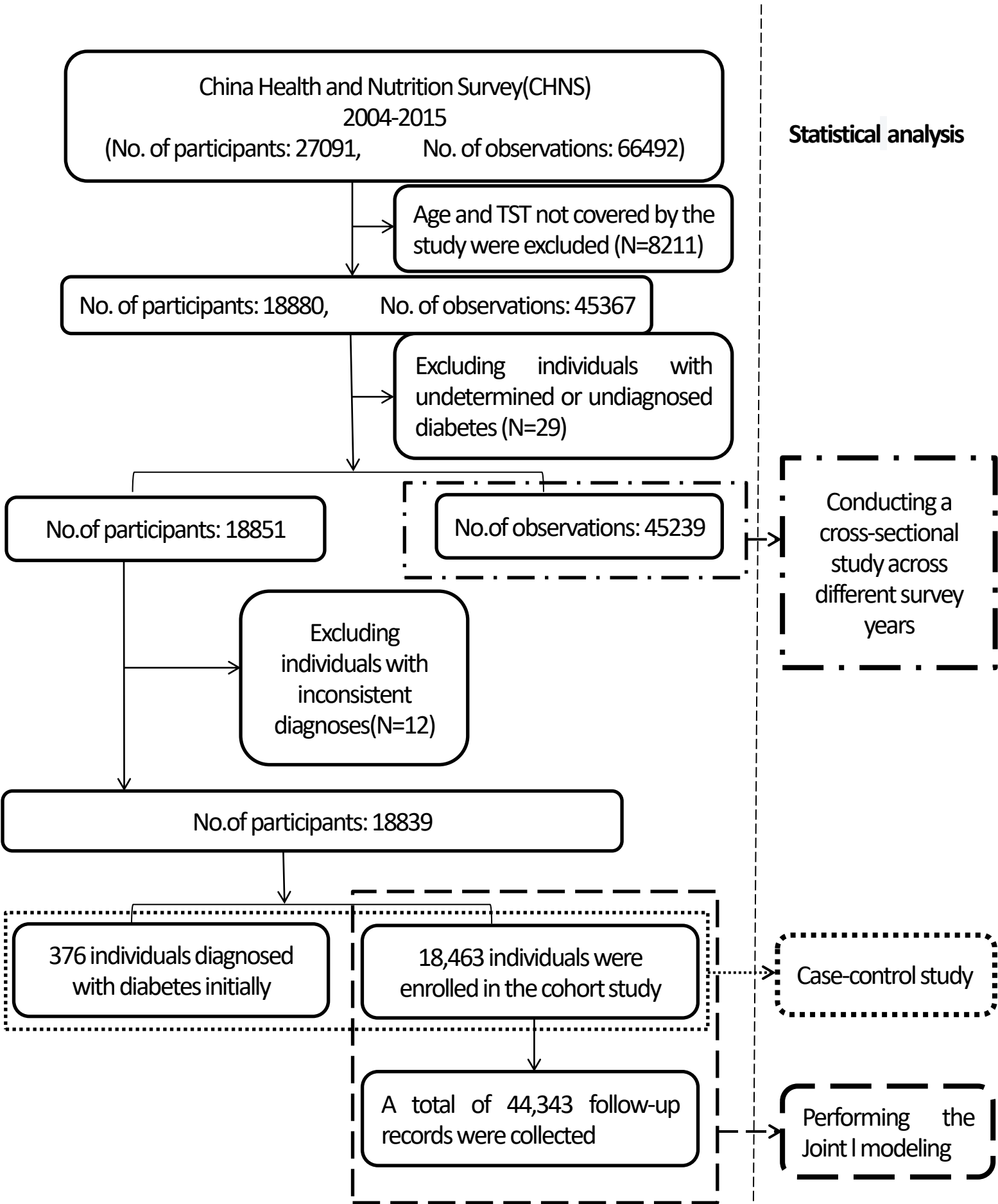

Figure 1 Flowchart for processing of research samples

Supplement: Supplementary file 1 [file DataSheet1.pdf]
