# Supplementary material for: Obesity and the accelerated decline in total sleep time increases the self-reported diagnoses of diabetes
Source: Front Endocrinol (Lausanne). 2025 May 12;16:1473892. doi: 10.3389/fendo.2025.1473892 (PMC12104077; doi:10.3389/fendo.2025.1473892)
Supplement: Supplementary file 2 [file Table1.docx]

Table S 1 Characteristics of Participant in the cohort study^¢^

|  | Observation year | | | | |
| --- | --- | --- | --- | --- | --- |
| All participants | 2004 (n = 8100) | 2006 (n =7917) | 2009 (n = 8137) | 2011 (n = 10455) | 2015 (n = 9734) |
| Sex(%) |  |  |  |  |  |
| Male | 3929(48.51) | 3804 (48.05) | 3910(48.05) | 4910 (46.96) | 4546 (46.70) |
| Age, Mean(SD) | 43.06 (12.03) | 44.09 (11.91) | 44.82 (12.16) | 45.62 (12.13) | 46.78 (12.02) |
| TST, Mean(SD) | 8.12(1.16) | 8.07(1.14) | 7.98(1.13) | 7.83(1.14) | 7.81(1.10) |
| Residence areas (%) |  |  |  |  |  |
| Urban | 2709(33.44) | 2593 (33.75) | 2715(33.37) | 4213(40.30) | 3680(37.81) |
| Education(%) |  |  |  |  |  |
| Low | 4780 (59.01) | 4220(53.30) | 4582(56.31) | 5227 (50.00) | 4766 (48.96) |
| Middle | 1803 (22.26) | 1840 (23.24) | 1748(21.48) | 2370(22.67) | 2386 (24.51) |
| High | 336(4.15) | 467 (5.90) | 486 (5.97) | 1411(13.50) | 1512 (15.53) |
| Unknown | 1181 (14.58) | 1390 (17.56) | 1321 (16.23) | 1447 (13.84) | 1070 (10.99) |
| Smoking(%) | 2682(33.11) | 2510(31.70) | 2578(31.68) | 3180(30.42) | 2623(26.95) |
| Alcohol drinking (%) | 2817(34.78) | 2691(33.99) | 2904(35.69) | 3792(36.27) | 2843(29.21) |
| Coffee or tea(%) | 3057(37.74) | 2771 (35.00) | 2939 (36.12) | 4354(41.65) | 2953(30.34) |
| Ethnicity |  |  |  |  |  |
| Han(%) | 7066(87.23) | 6924(87.46) | 7103(87.29) | 9454(90.43) | 8686(89.23) |
| PHD(%) | 426(5.26) | 449(5.67) | 473(5.81) | 641(6.13) | 549(5.64) |
| BMI (%) |  |  |  |  |  |
| NOB | 5128(64.50) | 4912 (62.90) | 4829 (60.14) | 5747(55.59) | 4740 (50.97) |
| Overweight | 2209(27.79) | 2269 (29.06) | 2447 (30.48) | 3358 (32.48) | 3224 (34.67) |
| Obesity | 613(7.71) | 628 (8.04) | 753 (9.38) | 1234 (11.94) | 1335 (14.36) |

^¢^Basic characteristics of the final modelling population
